# Potato Protein Peptides as a Serum‐Reducing Supplement for MDCK Cell Culture Medium

**DOI:** 10.1002/fsn3.72571

**Published:** 2026-11-05

**Authors:** Dandan Gao, Jiwen Wang, Huaxin Li, Qianmin Yang, Chenchen Wang, Yujie Cao, Junyao Mei, Xuankang Yang, Qianwen Bai, Gongtao Ding

**Affiliations:** ^1^ China‐Malaysia National Joint Laboratory, Biomedical Research Center Northwest Minzu University Lanzhou China; ^2^ College of Life Sciences and Engineering Northwest Minzu University Lanzhou China

**Keywords:** cell culture, MDCK cells, potato protein peptides, serum‐reducing supplement

## Abstract

Food‐derived peptides produced by enzymatic hydrolysis have attracted interest as functional biomaterials for cell culture. In this study, potato protein was hydrolyzed using Flavorzyme to prepare potato protein peptides (PPPs), which were characterized by degree of hydrolysis, SDS‐PAGE, free amino acid analysis, and LC–MS/MS‐based peptide identification. Among the 101 identified peptide sequences, 66 (65.35%) had molecular weights ranging from 500 to 1000 Da. And were subsequently evaluated for their effects on MDCK cell growth, metabolism, and cryopreservation. The optimal PPPs concentration for MDCK culture was 1 g/L. At passage 9, the PPPs + 5% NBS group showed the highest CCK‐8‐derived estimated cell density (9.11 × 10^4^ cells/mL), with a doubling time of 15.71 h and a specific growth rate of 0.0163 ± 0.0002 h^−1^. These values were significantly higher than those in the 5% newborn bovine serum (NBS) control (*p* < 0.05) and comparable to the 10% NBS control (*p* > 0.05). The PPPs + 5% NBS group exhibited glucose and lactate concentration profiles distinct from those of the 5% and 10% NBS controls during culture. In short‐term cryopreservation tests, passage‐10 cells maintained normal proliferation and morphology. PPPs supplementation was also associated with favorable post‐thaw cell recovery in serum‐containing cryopreservation media. Overall, these findings suggest that PPPs may serve as promising serum‐reducing supplements for MDCK cell culture and cryopreservation.

## Graphical abstract

Flavorzyme hydrolysis was used to prepare PPPs. Supplementation of the medium containing 5% NBS with 1 g/L PPPs supported MDCK cell proliferation and colony‐forming capacity during serial passaging; growth at passage 9 was comparable to that of the 10% NBS control. Furthermore, PPP‐based cryopreservation formulations containing residual NBS supported favorable post‐thaw cell recovery, indicating the potential of PPPs to partially reduce serum requirements in MDCK cell culture and cryopreservation.

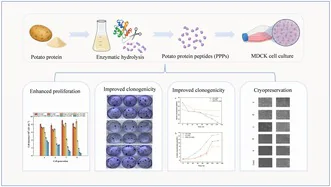

## 1 Introduction

Potato is an important global food crop and a major source of industrially processed protein resources (Devaux et al. 2014; Hussain et al. 2021). Potato starch processing generates protein‐rich side streams, such as potato fruit juice (PFJ), which has stimulated growing interest in the recovery and valorization of potato‐derived proteins (Fritsch et al. 2017; Fu et al. 2020; Taskila et al. 2017). Enzymatic hydrolysis can further improve the functional properties of potato proteins by generating low‐molecular‐weight peptides with potential biological activities. For instance, Rahimi et al. (2022) employed Alcalase to generate potato protein hydrolysates exhibiting both antioxidant capacity and α‐amylase inhibitory activity. Similarly, Chang et al. (2020) produced antioxidant‐active hydrolysates from potato protein isolate using a combination of bromelain, neutral protease, and Flavorzyme. Furthermore, accumulating evidence supports the application of potato protein hydrolysates in in vitro cell‐based assays and the development of biologics (Chang et al. 2020; Gao et al. 2021).

Serum widely used in mammalian cell culture because it provides growth factors, hormones, attachment proteins, and other nutrients required for cell proliferation. Nevertheless, growing concerns about the high economic burden, batch‐to‐batch variability, ethical considerations, and inherent risk of xenobiotic contamination associated with serum have catalyzed efforts to develop low‐serum and serum‐free culture strategies (Ho et al. 2021). Protein hydrolysates and peptide‐rich supplements have attracted particular attention because they can provide readily available nutrients and may also exert growth‐supporting biological effects (Kumar et al. 2021; Kwon et al. 2005; Obaidi et al. 2021). For example, Zhang et al. (2019) demonstrated that silk sericin hydrolysate, obtained from industrial silk‐processing waste streams, effectively replaces fetal bovine serum (FBS) in cultures of Chinese hamster ovary (CHO) cells and HeLa cells, supporting cell viability and proliferation rates comparable to or exceeding those achieved with standard FBS‐supplemented media. Similarly, Girón‐Calle et al. (2008) generated a bioactive peptide library via Alcalase‐ and Flavorzyme‐mediated hydrolysis of chickpea proteins and validated its efficacy as a serum replacement in THP‐1 monocyte cultures, where sustained proliferative capacity and phenotypic stability were maintained over multiple passages.

In this study, PPPs were evaluated as a serum‐reducing supplement for Madin–Darby canine kidney (MDCK) cell cultures. We investigated their effects on cell proliferation, growth kinetics, extracellular glucose and lactate profiles, clonogenic capacity, and post‐thaw recovery. The objective was to determine whether PPPs supplementation could support MDCK cell growth under reduced‐serum conditions and to provide an experimental foundation for the future development of more defined, low‐serum culture systems.

## 2 Materials and Methods

### 2.1 Materials

Potato protein (protein content was 86.7%) isolate was procured from Shaanxi Sinuote Bio‐Technology Co. Ltd. (Xi'an, China). Flavorzyme (activity: 200000 U/g, ≥ 95% purity) was obtained from Shandong Longtech Enzymes Co. Ltd. (Linyi, China). MDCK cells (ATCC CCL‐34) were acquired from the Gansu Technology Innovation Center for Animal Cell Culture, Northwest Minzu University (Lanzhou, China). Trypsin–EDTA solution (0.25%, *w/v*), Dulbecco's Modified Eagle Medium (DMEM) supplemented with L‐glutamine and sodium pyruvate, and NBS were purchased from Lanzhou Bailing Bio‐Tech Co. Ltd. (Lanzhou, China). CCK‐8 was sourced from Dalian Meilun Biotechnology Co. Ltd. (Dalian, China). Dimethyl sulfoxide (DMSO), crystal violet stain, and phosphate‐buffered saline (PBS, pH 7.4) were obtained from Beijing Solarbio Science & Technology Co. Ltd. (Beijing, China). Methanol (HPLC grade) was purchased from Sinopharm Chemical Reagent Co. Ltd. (Shanghai, China). Formaldehyde, trichloroacetic acid (TCA), formic acid, and acetonitrile were sourced from Merck KGaA (Germany). All other chemicals used in this study were of analytical grade.

### 2.2 Preparation of Potato Protein Peptides

Potato protein isolate (8.0 g) was dissolved in distilled water, and the final volume was adjusted to 100.0 mL to yield an 8% (*w/v*) substrate solution. The pH was carefully adjusted to 6.2 using 1 M HCl or NaOH. Flavorzyme (24,000 U/g, relative to substrate mass) was added to the pre‐warmed solution, and enzymatic hydrolysis was carried out at 50°C for 4 h in a thermostatically controlled water bath (DK‐8D, Shanghai Yiheng Scientific Instrument Co. Ltd., Shanghai, China). Reaction termination was achieved by heating the mixture to 90°C for 15 min. Subsequently, the denatured enzyme and insoluble aggregates were removed by centrifugation at 8000 r/min for 15 min (LC‐6 M, Shanghai Centrifuge Research Institute Co. Ltd., Shanghai, China). The clarified supernatant was then subjected to ultrafiltration through a 5‐kDa molecular weight cutoff to isolate low‐molecular‐weight peptides. The resulting PPPs solution was immediately frozen at −80°C and subsequently lyophilized (LGJ‐20F, Beijing Songyuan Huaxing Science and Technology Development Co. Ltd., Beijing, China). The dried PPPs powder was stored under desiccated conditions at −20°C prior to use (Echers et al. 2023; Sung et al. 2025).

### 2.3 Determination of the DH

The total nitrogen (TN) content in potato protein was determined using the Kjeldahl method, while ammonia nitrogen (AN) was measured via formaldehyde titration (Jung et al. 2003; Morais et al. 2013; Zhou et al. 2012). The procedure is as follows: Accurately pipette 10.00 mL (V_0_) of an 80 mg/mL potato protein hydrolysate and titrate with 0.10 mol/L NaOH solution to pH 8.2. Then, add 10.0 mL of neutral formaldehyde solution (20%, *v/v*), pre‐adjusted to pH 8.2. After thorough mixing, continue titrating with 0.10 mol/L NaOH standard solution until the pH reaches 9.20. Record the volume of NaOH consumed (V_1_). Perform a blank titration using 10.00 mL of deionized water instead of the sample, following the same procedure, and record the blank volume (V_2_). Calculate the degree of hydrolysis (DH) using the following formula, with the ratio of AN to TN as the standard:

(1) $$\mathrm{DH}\%=\frac{\text{AN}}{\mathrm{TN}}\times 100$$

(2) $$\text{AN}\%=\frac{\left(V_{1}-V_{2}\right)\times 1.4}{0.2\times V_{0}}$$

### 2.4 Determination of Molecular Weight of PPPs

The molecular weight of PPPs was analyzed using Tris‐SDS‐PAGE gel electrophoresis. Samples were diluted to a final concentration of 1 mg/mL. The sample buffer was prepared by mixing 0.25 M Tris–HCl (pH 6.8), 10% SDS, 0.5% bromophenol blue, 50% glycerol, and 5% β‐mercaptoethanol. The sample buffer and sample solution were thoroughly mixed at a 1:4 ratio. The mixture was denatured by heating at 100°C for 10 min, followed by electrophoresis at 80 V for 2 h under low‐temperature conditions. The gel was stained with KEMAS Brilliant Blue G‐250 reagent, then decolorized and visualized (Mohanty et al. 2021).

### 2.5 Detection of Free Amino Acids in PPPs

Refer to the method of Xiang et al. (2025). Dissolve PPPs in pure water. Centrifuge at 1200 r/min for 15 min to remove proteins. Add an equal volume of 10% TCA to the supernatant and mix thoroughly. Allow the mixture to stand at 4°C for 2 h, then filter through a 0.22 μm filter. Determine free amino acids using an amino acid analyzer (Model L‐8900, Hitachi Co., Japan) equipped with a cation exchange column. Nitrogen‐derived amino acids were detected at wavelengths of 570 nm and 440 nm. The sample volume was 20 μL, with the column temperature set at 135°C and the flow rate maintained at 0.35 mL/min.

### 2.6 Mass‐Spectrometric Identification of PPPs

Peptide‐sequence analysis of PPPs was performed using a liquid chromatography‐tandem mass spectrometer (Q‐Exactive Plus, Thermo Fisher Scientific, Bremen, Germany) (Qian et al. 2021). Peptides were separated on a nano‐reversed‐phase C18 column (75 μm × 250 mm, 2 μm). Mobile phase A consisted of 98% water, 0.1% formic acid, and 2% acetonitrile; mobile phase B consisted of 2% water, 0.1% formic acid, and 98% acetonitrile. Gradient elution was performed as follows: 0.0 min, 5% B; 0.5 min, 8% B; 0.6 min, 12% B; 25 min, 30% B; 30 min, 35% B; 30.5 min, 80% B; 38.5 min, 5% B; 50 min, 5% B.

Mass‐spectrometric data were acquired in positive‐ion mode with a precursor ion scan range of m/z 350–1800. Full‐scan MS resolution was set to 70,000 at m/z 200, AGC target of 3e6, and maximum injection time of 50 ms. In data‐dependent acquisition mode, the top 20 most intense precursor ions from each full‐scan event were selected for MS/MS fragmentation. MS2 resolution was 17,500 at m/z 200, AGC target of 1e5, and maximum injection time of 45 ms. Fragmentation was achieved by higher‐energy collisional dissociation with an isolation window of 2.0 m/z and a normalized collision energy of 28.

PEAKS Studio 10.0 software was used for mass‐spectrometric database searching. The protein database was downloaded from the UniProt Protein Database for *Solanum tuberosum* (potato), containing 75,051 protein entries. Peptide sequences within PPPs were identified by matching experimental spectra against this potato protein database.

### 2.7 MDCK Cell Culture

Frozen MDCK cells were rapidly thawed in a 37°C water bath and transferred to 9 mL of pre‐warmed complete medium consisting of DMEM supplemented with 10% NBS. After centrifugation at 1000 r/min for 5 min at room temperature, the cells were resuspended in fresh complete medium, seeded into T25 flasks, and incubated at 37°C in a humidified atmosphere containing 5% CO_2_ (Model 31, Thermo Fisher Scientific, Waltham, MA, USA). After 8–10 h, non‐adherent cells and debris were removed by washing twice with PBS, followed by replacement with fresh complete medium.

Cells were routinely subcultured at 80%–90% confluence. Briefly, the monolayer was washed once with PBS and treated with 2 mL of 0.25% (*w/v*) trypsin–EDTA for 2–3 min at 37°C. Cell detachment was monitored using an inverted microscope (IX51, Olympus Corporation, Tokyo, Japan), and trypsinization was terminated with an equal volume of complete medium. The cell suspension was gently dispersed and reseeded into new culture vessels at the required split ratio for subsequent experiments (Cai et al. 2024).

### 2.8 Effect of PPPs Concentration on MDCK Cell Proliferation

To evaluate the effect of PPPs concentration on MDCK cell proliferation (Mazurkova et al. 2009), lyophilized PPPs were added to DMEM supplemented with 5% NBS to final concentrations of 0.5, 1.0, 2.0, 3.0, 4.0, and 5.0 g/L, followed by sterile filtration through a 0.22 μm membrane. MDCK cells were seeded in 12‐well plates at 1 × 10^5^ cells/mL and cultured in the corresponding PPPs‐supplemented media. DMEM containing 10% NBS and 5% NBS served as control media. Cells were maintained at 37°C in a humidified atmosphere containing 5% CO_2_. After 48 h, viable cell density was determined using an automated cell counter (Countstar, Shanghai Ruiyu Biotechnology Co. Ltd., Shanghai, China), followed by subculture. Cell morphology and adhesion were monitored by inverted microscopy. The optimal PPPs concentration was determined by comparing cell density, population doubling time, and morphological characteristics among the experimental and control groups.

### 2.9 Evaluation of MDCK Cell Proliferation

A reference calibration curve relating CCK‐8 absorbance to cell number was established using MDCK cells cultured in DMEM supplemented with 10% NBS. Cells were seeded in 96‐well plates at 5 × 10^3^–3 × 10^4^ cells/well in 100 μL medium. After 24 h, 10 μL of CCK‐8 reagent was added, followed by incubation for 2.5 h at 37°C, and absorbance was measured at 450 nm using a microplate reader (MK3, Thermo Fisher Scientific, Waltham, MA, USA).

For proliferation analysis across passages 3, 5, 7, and 9, MDCK cells were assigned to three groups: DMEM + 10% NBS, DMEM + 5% NBS, and DMEM + 5% NBS + 1 g/L PPPs. Cells in logarithmic growth phase were seeded into 96‐well plates at 5 × 10^3^ viable cells/mL in 200 μL/well. At 0, 1, 2, 3, 4, 5, and 6 days after seeding, CCK‐8 absorbance was measured as described above.

Absorbance values were converted to estimated cell‐density values using the reference calibration curve established under the 10% NBS condition. The resulting estimated growth curves were used to calculate the maximum estimated cell density, doubling time (Liu et al. 2025), and specific growth rate (Trabelsi et al. 2006). Because condition‐specific calibration curves were not established, these parameters were interpreted as approximate indicators of relative growth kinetics rather than direct absolute measurements.

(3) $$D=T\times \frac{X}{\log_{2}Y}$$

where D is the doubling time, *X* is the number of cells initially inoculated, *Y* is the number of cells on the day before the maximum cell proliferation density, and *T* is the incubation time.

(4) $$S=\frac{\text{In}\left(\frac{X_{n}}{X_{n-1}}\right)}{t_{n}-t_{n-1}}$$

where *S* is the specific growth rate, *X* is the live cell density, *t* is the culture time, *n* and *n* − 1 are the 2 sampling and counting time points.

### 2.10 Cell Cloning Rate

Following the grouping conditions described in Section 2.5, MDCK cells were seeded at a density of 50 cells per well in 6‐well plates, with 4 mL of complete medium added to each well. Cells were incubated at 37°C in a humidified 5% CO_2_ incubator for 7–14 days, with the medium refreshed every 2–3 days using pre‐warmed complete medium. At the end of the culture period, the medium was aspirated and adherent cells were gently rinsed once with PBS. Subsequently, 1 mL of ice‐cold methanol was added to each well for fixation at room temperature for 10 min. After removal of the fixative, 1 mL of 0.5% (*w/v*) crystal violet solution was added to each well and incubated for 20–30 min at room temperature. After staining, discard the staining solution. Manually count the cell colony clusters and calculate the cell cloning rate (Z. Liu et al. 2023).

(5) $$\text{Cell cloning rate}\%=\frac{\text{Number of colonies}}{\text{Number of}\;\text{inoculated cells}}\times 100$$

### 2.11 Measurement of Glucose and Lactate Concentrations in MDCK Cell Cultures

According to the grouping conditions described in Section 2.5, MDCK cells were seeded at a density of 3 × 10^4^ viable cells per well in 24‐well plates (final volume per well: 3 mL). Cells were cultured at 37°C in a humidified 5% CO_2_ incubator for 7 days. Samples were collected every 24 h post‐seeding: 1 mL of culture supernatant was aspirated from each well, centrifuged at 3000 r/min for 5 min at 4°C, and the clarified supernatant was collected. Glucose and lactate (Carinhas et al. 2016) concentrations in the supernatant were quantified using a multiparameter clinical chemistry analyzer (Hitachi 7020, Hitachi High‐Technologies Corporation, Tokyo, Japan). During the culture period, 1 mL of pre‐warmed corresponding medium was replenished into each well immediately after sampling to maintain constant culture volume and nutrient homeostasis.

### 2.12 Effect of PPPs on Cell Growth Kinetics

Based on the MDCK cell growth data, nonlinear regression fitting was performed using MATLAB R2022b (MathWorks, Natick, MA, USA) to estimate the model parameters, including the maximum cell density (*X*_max_) and maximum specific growth rate (*μ*_max_). The logistic growth equation (Xu 2020) was used as a descriptive model to characterize the overall growth pattern of MDCK cells under the different culture conditions. Model fit was evaluated using the coefficient of determination (*R* ^2^).

(6) $$\frac{dX}{dt}=\mu {\frac{X}{X_{\max}}}_{\max}$$

where *X* is the cell density, *μ*_max_ is the maximum specific growth rate, and *X*_max_ is the maximum cell growth density.

When the initial condition *t* = 0, the cell density *X* is equal to the initial cell density *X*_0_. Solving the equation of Equation (6) leads to Equation (7):

(7) $$X=\frac{X_{0}X_{\max}e^{\mu_{\max}t}}{X_{\max}-X_{0}+X_{0}e^{\mu_{\max}t}}$$

The cell specific growth rate and the maximum cell growth density were obtained from the above equations.

### 2.13 Effect of PPPs Pretreatment on Short‐Term Cryopreservation and Recovery of MDCK Cells

MDCK cells pre‐cultured for 48 h in medium containing the optimal PPPs concentration were harvested with 0.25% trypsin–EDTA and centrifuged at 1000 r/min for 5 min. The cell pellet was resuspended in cryopreservation medium containing 10% DMSO, 20% NBS, and 70% DMEM (*v/v*) to a final density of 2 × 10^6^ viable cells/mL. Aliquots (1 mL) were transferred to cryovials, cooled at approximately −1°C/min to −80°C overnight using a controlled‐rate freezing container (Mr. Frosty, Thermo Fisher Scientific), and then transferred to the vapor phase of liquid nitrogen (Miyamoto et al. 2018).

After 1 week, cells were rapidly thawed at 37°C and immediately diluted with 5 mL of pre‐warmed complete medium. Following centrifugation at 1000 r/min for 5 min, the cell pellet was resuspended in 2 mL of fresh complete medium and seeded into T25 flasks at 1 × 10^4^ cells/cm^2^. Cells were cultured at 37°C in a humidified atmosphere containing 5% CO_2_, and post‐thaw morphology, attachment, and proliferation were evaluated by inverted microscopy after 24 h.

### 2.14 Application of PPPs for Serum‐Reducing Additive in Cell Cryopreservation

To evaluate whether PPPs could serve as serum‐reducing additives for NBS in cryopreservation media, six compositionally distinct formulations were prepared (Table 1). Cryopreservation and post‐thaw recovery procedures for each experimental group strictly adhered to the standardized protocols detailed in Section 2.13. Cell viability was assessed using microscopy.

**TABLE 1:** Composition of cryopreservation solutions for each group.

Group	Cryopreservation solution volume ratio DMSO: NBS: DMEM: PPPs (%)
Control group	10:20:70:0
A group	10:0:0:90
B group	10:5:0:85
C group	10:10:0:80
D group	10:15:0:75
E group	10:20:0:70

*Note:* PPPs concentrations in the table are 1 g/L (PPPs dissolved in DMEM).

### 2.15 Statistical Analysis

All experiments were performed in three independent biological replicates (*n* = 3), and data are presented as mean ± standard deviation (SD). Statistical analyses were performed using SPSS Statistics 27.0 and OriginPro 2021b. Normality and homogeneity of variance were assessed using the Shapiro–Wilk and Levene's tests, respectively. One‐way ANOVA followed by Tukey's multiple‐comparison test was used for datasets involving a single experimental factor, whereas two‐way ANOVA followed by Tukey's multiple‐comparison test was used for datasets involving treatment × passage or treatment × culture time. A value of *p* < 0.05 was considered statistically significant.

## 3 Results

### 3.1 DH of Potato Protein

The DH of potato protein reached 42.83%.

### 3.2 Molecular Weight of PPPs

Figure 1 shows that when comparing lane B to lane A, the high molecular weight bands in lane B lighten and disappear, while diffuse or new bands appear in the low molecular weight region, which is direct evidence that the protease has successfully cleaved the large protein molecules into smaller peptide fragments. Lane B shows the characteristic hydrolysis product (17 kDa), a continuous “trailing” or “diffuse” band is present, indicating that the hydrolysis was complete and a wide range of peptides with a wide range of molecular weights were produced. The significant molecular weight bands of potato proteins were in the range of 34 kDa‐72 kDa and greater than 17 kDa, and the potato proteins were hydrolyzed to a variety of small molecules with molecular weight distributions less than 17 kDa after sufficient enzymatic hydrolysis. The results showed that the proteins in PPPs were completely hydrolyzed, and the molecular weight ranges were all less than 17 kDa.

**FIGURE 1:**
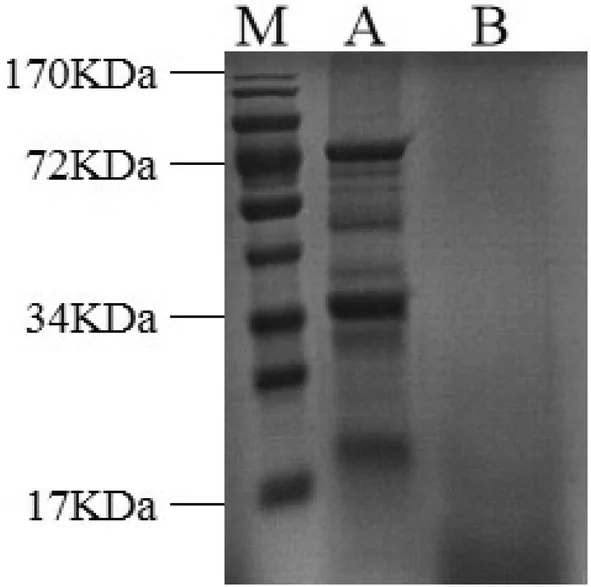
SDS‐PAGE gel electrophoresis analysis. M: Protein molecular‐weight marker; A: Native potato protein; B: PPPs.

### 3.3 Free Amino Acids in PPPs

According to the results in Table 2, PPPs contain a total of 24 free amino acids. The total amino acids (TAA) amounted to 38.44%. The essential amino acid (EAA) content amounted to 13.22%, accounting for 34.4% of the total. The non‐essential amino acid (NEAA) content was 25.22%, accounting for 65.9% of the total.

**TABLE 2:** Content of free amino acids in PPPs.

Category	Amino acid	(w/w%)
EAA	Lys	0.031
	Trp	0.708
	Phe	1.644
	Met	0.628
	Thr	1.673
	Ile	2.281
	Leu	3.909
	Val	2.345
NEAA	Gly	1.063
	Ala	1.857
	Pro	2.972
	Tyr	3.909
	Ser	2.339
	Cys	0.269
	AspNH_2_	0.168
	Asp	1.233
	GluNH_2_	2.511
	Glu	2.393
	His	2.404
	Cit	0.117
	Orn	0.060
	P‐Ser	0.309
	Arg	0.993
	g‐ABA	2.628
TAA		38.44
EAA/TAA		34.4
NEAA/TAA		65.9

### 3.4 Peptide Sequence Analysis of PPPs

As shown in Table 3, a total of 101 peptide sequences were identified from the PPPs sample. Based on the molecular weight distribution of the identified peptides, 2 peptides exhibited molecular weights above 3000 Da, 2 peptides ranged from 2000 to 3000 Da, 31 peptides ranged from 1000 to 2000 Da, and 66 peptides were within the range of 500–1000 Da. Among them, 15 peptides were distributed in the 900–1000 Da range. The mass spectrometry identification results demonstrated that PPPs were predominantly composed of short peptides with molecular weights mainly distributed between 500 and 1000 Da.

**TABLE 3:** Identification of PPPs peptide segments.

No.	Peptide sequence	Molecular mass (Da)	Length (aa)	m/z
1	ASGSGVP	573.275	7	574.287
2	TAAASTS	607.281	7	608.286
3	TGLVGAP	613.343	7	614.348
4	PGPSGLP	623.327	7	624.337
5	GIGGICG	632.295	7	633.303
6	EEGGVGP	643.281	7	644.288
7	HPGFGGP	667.307	7	668.316
8	VPGGTGGK	671.360	8	672.363
9	SAADNGF	680.276	7	681.277
10	TPIGGFP	687.359	7	688.369
11	TLSGTIP	687.380	7	688.385
12	SAMIGGY	697.310	7	698.318
13	TVRGGLP	698.407	7	699.411
14	IMSSAVP	703.357	7	704.363
15	NVMAPGH	724.332	7	725.342
16	ETTTSPP	731.333	7	732.344
17	TPDGPVF	731.349	7	732.358
18	LPSATFP	731.385	7	732.399
19	SPIDVIT	743.406	7	744.415
20	LGTDAQF	750.354	7	751.363
21	TATTNFP	750.354	7	376.187
22	EPISTGGP	756.365	8	757.378
23	IPGFDIP	757.401	7	758.411
24	FSHDAGK	760.350	7	761.353
25	PETQPPP	765.354	7	766.365
26	SIIDGIR	772.444	7	387.230
27	FPGDEIP	773.359	7	774.364
28	DPEGLFP	773.359	7	774.367
29	LCFAADP	792.347	7	793.358
30	IARQFGI	803.465	7	402.741
31	SPTRHPI	806.439	7	404.228
32	YSSIKLP	806.453	7	404.234
33	RSGNLGDP	815.377	8	816.378
34	QSQWAVP	815.381	7	816.380
35	FDSLPTH	815.381	7	816.389
36	LNQNLGC	817.375	7	818.383
37	LPGDNVGF	817.397	8	818.409
38	DPLGRFQ	831.423	7	832.432
39	VPPFDYP	833.395	7	834.404
40	KDYTDPP	834.375	7	835.389
41	TVDDFVF	841.385	7	842.394
42	SLDTFIF	841.422	7	842.426
43	PLIHGPIP	842.501	8	422.259
44	EKELLNI	857.485	7	858.492
45	PFLPFPF	863.458	7	864.468
46	LEAFAIDS	864.422	8	865.432
47	HDTSLPIP	878.449	8	440.228
48	FPPSSFVV	878.453	8	879.457
49	YSSSSQPQ	882.371	8	883.381
50	YPPGPYPP	886.422	8	887.422
51	LEPEFRP	886.454	7	444.235
52	KMSYPGAH	889.411	8	890.410
53	ASGGQASHAL	898.414	10	450.217
*54	YVCKISH	905.442	7	906.453
*55	ADSFQPPF	907.407	8	908.410
*56	SLSPDGVAY	907.428	9	908.429
*57	KFAEAAYN	913.418	8	914.424
*58	LQQELKC	917.464	7	918.472
*59	EEDAPLRP	925.450	8	463.732
*60	KLKVKHGK	936.623	8	469.318
*61	SQWIQKGL	959.507	8	480.762
*62	CPAFPLCP	960.419	8	961.426
*63	GQFGVGFYS	960.434	9	961.438
*64	LLQLPHNF	980.544	8	491.2799
*65	TPIGGFPHY	987.481	9	494.748
*66	SLFMPNGLP	990.484	9	496.247
*67	EAELGPEPR	996.487	9	499.252
*68	ANGGANTAAPGT	1000.457	12	1001.462
69	TACQQQGTE	1022.397	9	1023.399
70	VPTVDVSVVD	1028.538	10	515.278
71	QLIVVPQNF	1056.596	9	529.305
72	SEYPPLGRF	1064.529	9	533.273
73	NTYAIDTQF	1072.471	9	1073.473
74	VPRYFPFC	1084.516	8	543.267
75	TLGNFVKATF	1096.591	10	549.305
76	PLSVYLLSMP	1118.604	10	560.308
77	ECVHDGWGVA	1128.465	10	565.241
78	VPTVDVSVVDL	1141.623	11	571.818
79	AGIGSDHVDLKA	1181.604	12	591.811
80	FSEYPPLGRF	1211.597	10	606.807
81	FRATRSYTNF	1261.620	10	631.815
82	DPKPIPGDWNGA	1265.604	12	633.811
83	TLVDNQSTLPVP	1282.676	12	642.348
84	VEVLTLSMADHP	1310.654	12	656.330
85	DFFTNCGKIRF	1403.665	11	702.841
86	FPPDYPFKPPKV	1430.759	12	477.929
87	NHPGQIGNGYAPVL	1436.704	14	719.361
88	SPCGEGTNTWDRF	1525.625	13	763.820
89	RNSWGTNWGEDGY	1541.617	13	771.811
90	RNSWGTNWGEDGY	1542.601	13	772.303
91	SIVVIGHVDSGKSTTT	1599.846	16	800.932
92	LLLTAGIGSDHVDLKA	1621.903	16	811.961
93	AIVQVDAAPFKQWY	1634.845	14	818.428
94	EGKEEDQELVIEDN	1645.731	14	823.869
95	VEEEEEEPEPVKEEE	1828.773	15	915.398
96	WVTHQYSRVPVFEQR	1930.980	15	387.202
97	LPFSPDQEVEVAKEPGQP	1965.968	18	656.325
98	MESSKEENENLGKKAGEAEG	2137.932	20	1069.976
99	IFQALCNYGELIGQLDLMGSERPQ	2754.288	24	689.583
100	DTEDKSAKRKKKQDTYRTLLQSGEGSDGNEDDDQ	3857.778	34	1286.921
101	TQGEIISCNLDARRCFSMGSFQYVVGDSELQIAL	3867.764	34	774.559

### 3.5 Post‐Thaw Recovery

MDCK cells were cultured for 48 h post‐thaw, and the viable cell density was determined to be 8.31 × 10^5^ cells/mL with a viability of 92.8%, as assessed by trypan blue exclusion assay. Subsequently, MDCK cells were serially passaged for three consecutive generations at a seeding density of 5 × 10^4^ cells/cm^2^, maintaining > 95% viability throughout all passages, thereby confirming robust recovery and proliferative capacity following cryopreservation.

### 3.6 Effect of PPPs on MDCK Cell Density

As shown in Figure 2, MDCK cells at passage 7 exhibited concentration‐dependent morphological responses to PPPs after 48 h of culture. Cells cultured in 10% NBS and 5% NBS showed similar epithelial morphology. Supplementation with 0.5–2 g/L PPPs maintained relatively compact cell arrangement, clear cell boundaries, and good monolayer formation. In contrast, cells exposed to 3–5 g/L PPPs showed progressively more pronounced morphological alterations, including elongation, aggregation, reduced monolayer coverage, and an apparent decrease in cell abundance.

**FIGURE 2:**
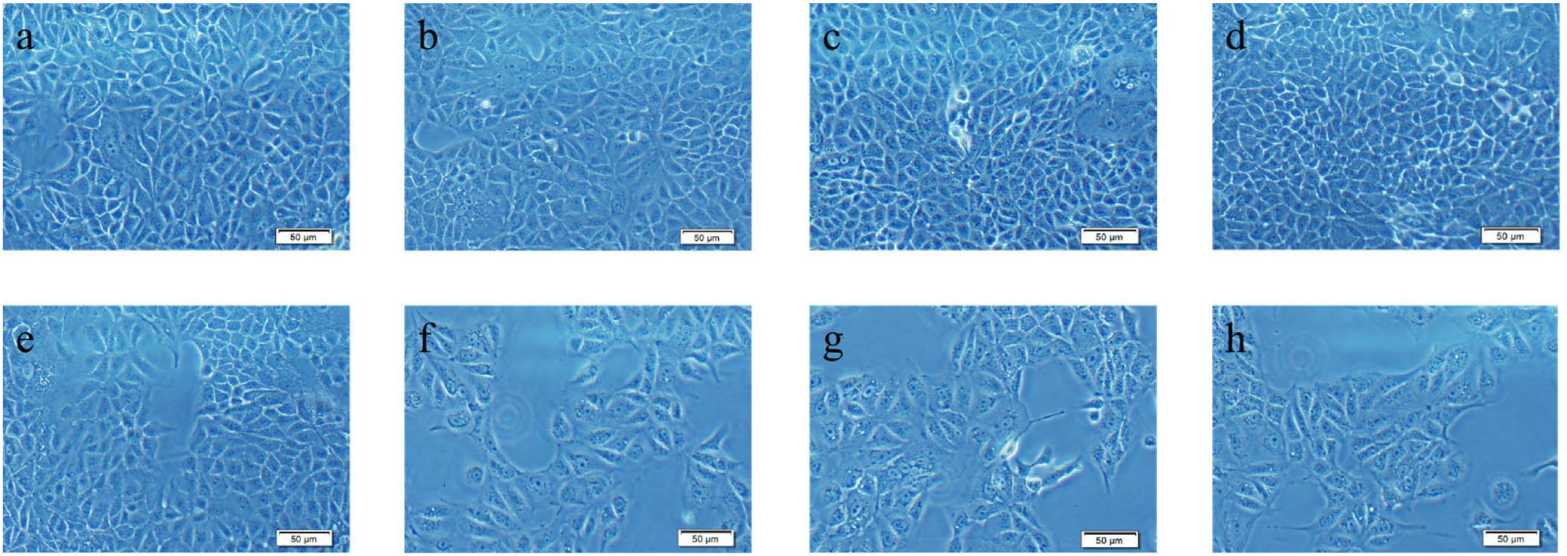
The growth of the 7th generation MDCK adherent cells at 48 h. (a):10% NBS; (b):5% NBS; (c):0.5 g/L PPPs + 5% NBS; (d) 1 g/L PPPs + 5% NBS; (e) 2 g/L PPPs + 5% NBS; (f) 3 g/L PPPs + 5% NBS; (g) 4 g/L PPPs + 5% NBS; (h) 5 g/L PPPs + 5% NBS.

As shown in Figure 3, viable cell density was also affected by PPPs concentration during serial passage. Cells cultured with 0.5 or 1 g/L PPPs maintained relatively high cell densities, whereas supplementation with 2–5 g/L PPPs resulted in progressively lower cell densities as the PPPs concentration increased. Among the concentrations tested, 1 g/L PPPs showed the most favorable effect on MDCK cell growth during later passages, while higher concentrations, particularly 3–5 g/L, were associated with reduced cell density and altered cell morphology. These results indicate a clear concentration‐dependent effect of PPPs on MDCK cell growth, with 1 g/L identified as the optimal concentration under the present experimental conditions.

**FIGURE 3:**
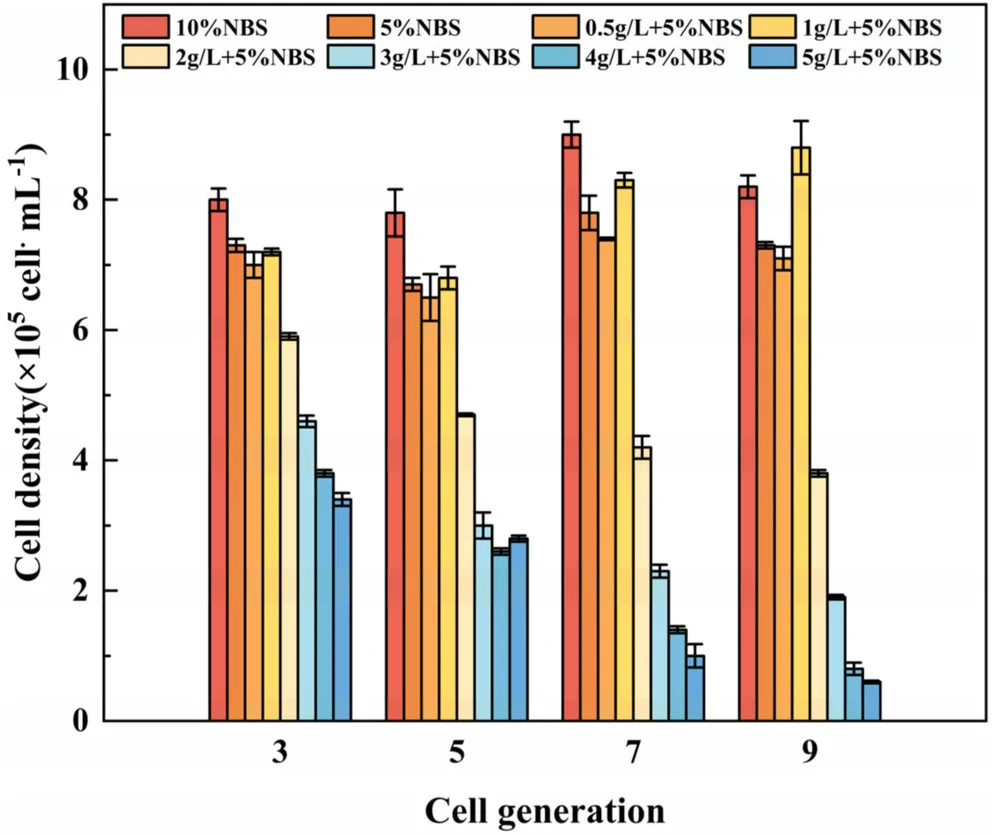
Comparison of MDCK cell density in different generations.

### 3.7 Effect of PPPs on CCK‐8‐Derived Growth Parameters

As shown in Figure 4 and Table 4, at passage 3, both control and PPP‐treated experimental groups reached peak estimated cell density at 144 h. The rank order of maximal estimated cell densities was: 10% NBS control > PPPs + 5% NBS experimental group > 5% NBS control. At 144 h, the exponential growth rate of the 10% NBS control group was 0.0127 ± 0.0003 h^−1^—significantly higher than those of both the 5% NBS control and the PPPs + 5% NBS experimental group (*p* < 0.05). Concurrently, the doubling time of the 10% NBS control group was the shortest at 20.81 h, whereas the doubling time of the PPPs + 5% NBS group was 22.77 h—comparable to that of the 10% NBS control and significantly shorter than that of the 5% NBS control group.

**FIGURE 4:**
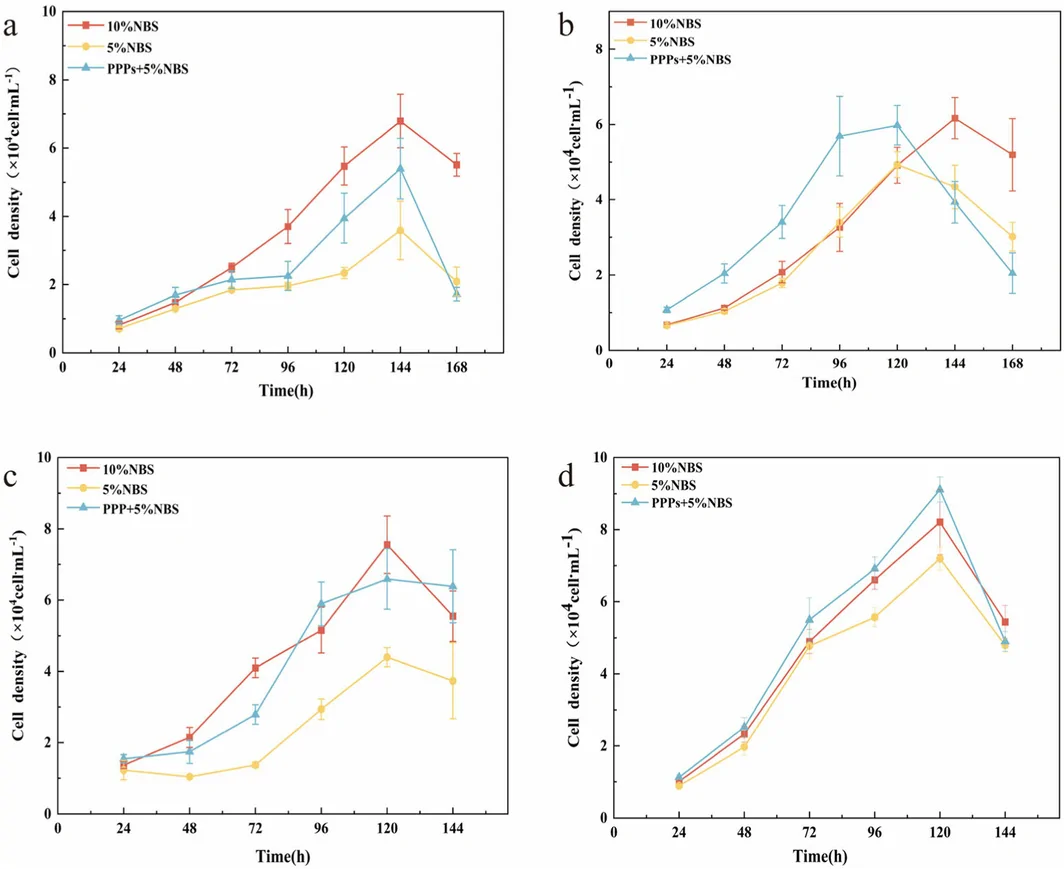
Cell growth curves. (a) Cell growth of the 3rd generation; (b) Cell growth of the 5th generation; (c) Cell growth of the 7th generation; (d) Cell growth of the 9th generation.

**TABLE 4:** CCK‐8‐derived estimated maximum cell density, doubling time, and specific growth rate of MDCK cells at passages 3, 5, 7, and 9.

Generation	Groups	Estimated maximum cell density	Doubling time	Specific growth rate
		(10^4^ cells/mL)	(h)	(h^−1^)
3rd	5% NBS	4.01	26.41	0.0110 ± 0.0005
	10% NBS	6.79	20.81	0.0127 ± 0.0003
	PPPs + 5% NBS	5.39	22.77	0.0119 ± 0.0005
5th	5% NBS	4.93	18.91	0.0140 ± 0.0002
	10% NBS	6.17	19.26	0.0141 ± 0.0003
	PPPs + 5% NBS	5.98	16.56	0.0147 ± 0.0003
7th	5% NBS	4.39	19.72	0.0136 ± 0.0002
	10% NBS	7.55	16.91	0.0156 ± 0.0004
	PPPs + 5% NBS	6.59	16.35	0.0151 ± 0.0004
9th	5% NBS	7.19	16.56	0.0154 ± 0.0004
	10% NBS	8.21	15.88	0.0159 ± 0.0001
	PPPs + 5% NBS	9.11	15.71	0.0163 ± 0.0001

At passage 5, the estimated cell densities of both the PPPs + 5% NBS experimental group and the 5% NBS control group peaked at 120 h. In contrast, the 10% NBS control group attained the highest maximum CCK‐8‐derived cell‐density estimate among all groups at 144 h—a delay consistent with transient metabolic adaptation rather than growth inhibition. Concurrently, the doubling time of the PPPs + 5% NBS group was significantly shorter at 16.56 h, compared to 19.26 h for the 10% NBS control group and 18.91 h for the 5% NBS control group. The specific growth rate of the PPPs + 5% NBS group was 0.0148 ± 0.0002 h^−1^—significantly higher than those of both the 10% NBS and 5% NBS control groups.

At passage 7, following 120 h of culture, both control and PPPs + 5% NBS experimental groups reached peak estimated cell density. Furthermore, the 10% NBS control and PPPs + 5% NBS experimental groups exhibited statistically significant increases in viable cell density relative to the 5% NBS control group (*p* < 0.05). At this time point, the doubling time of the PPPs + 5% NBS group was 16.35 h—statistically indistinguishable from that of the 10% NBS control group (16.42 h) but significantly shorter than that of the 5% NBS control group (18.73 h). The specific growth rate of the PPPs + 5% NBS group was 0.0151 ± 0.0004 h^−1^, significantly exceeding that of the 5% NBS control group (*p* < 0.05) and comparable to that of the 10% NBS control group (0.0150 ± 0.0003 h^−1^).

At passage 9, following 120 h of culture, both control and PPPs + 5% NBS experimental groups reached peak estimated cell density; notably, the 10% NBS control and PPPs + 5% NBS experimental groups significantly exceeded the 5% NBS control group. Concurrently, doubling times were 16.56 h for the 5% NBS control group, 15.88 h for the 10% NBS control group, and 15.71 h for the PPPs + 5% NBS experimental group. Corresponding specific growth rates were 0.0154 ± 0.0004 h^−1^, 0.0159 ± 0.0001 h^−1^, and 0.0163 ± 0.0002 h^−1^, respectively. The PPPs + 5% NBS group exhibited the shortest doubling times and the highest specific growth rate among all groups—demonstrating a consistent enhancement in proliferative capacity relative to both control groups.

### 3.8 Cell Cloning Rate

Based on the colony formation assay in 6‐well plates (Figure 5), the cell cloning rates of the 10% NBS control, 5% NBS control, and PPPs + 5% NBS experimental groups were 43.6%, 39.8%, and 45.1%, respectively. The PPPs + 5% NBS group exhibited an increase in cell cloning rates relative to both control groups. These results suggest that PPPs supplementation under a 5% NBS background modestly increased the clonogenic capacity of MDCK cells under the tested conditions.

**FIGURE 5:**
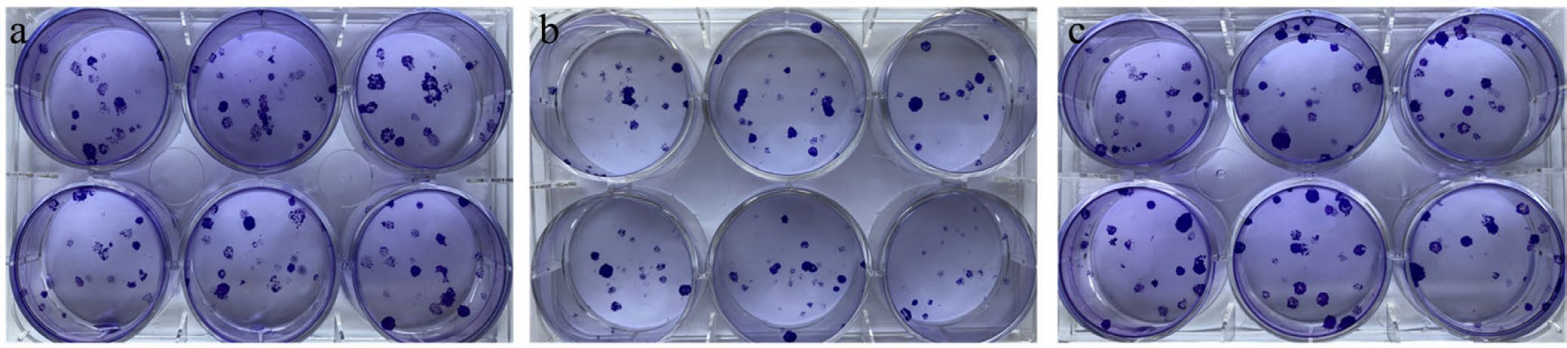
Colony formation rate of MDCK cells under different culture conditions. (a) 10% NBS; (b) 5% NBS; (c) 1 g/L PPPs + 5% NBS.

### 3.9 Effects of PPPs on Glucose and Lactate Concentrations in MDCK Cell Cultures

As shown in Figure 6, glucose concentrations in the culture medium generally decreased over time in all groups, whereas lactate concentrations increased progressively. Because 1 mL of culture medium was removed and replaced with an equal volume of fresh medium at each sampling point, the measured values represent time‐point‐specific concentrations rather than cumulative glucose consumption or lactate production.

**FIGURE 6:**
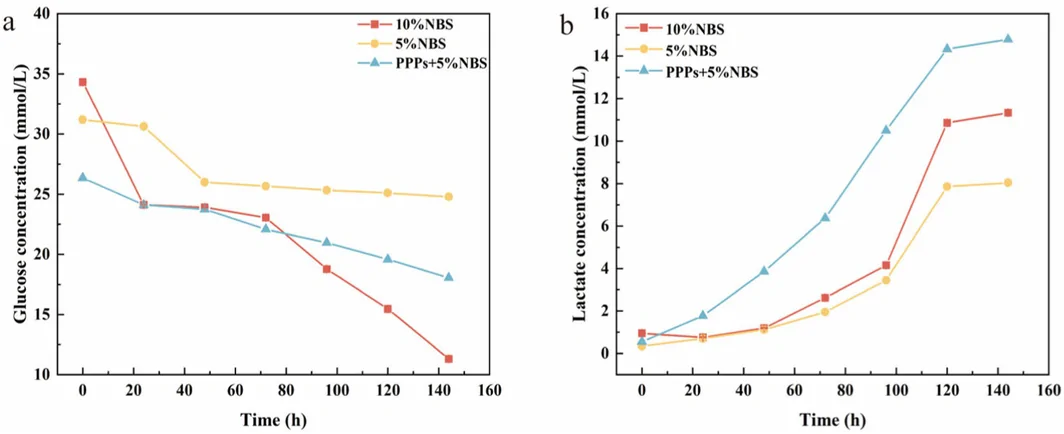
Changes in glucose (a) and lactate (b) concentrations in MDCK cell culture medium under different culture conditions.

Initial glucose concentrations differed among the 5% NBS, 10% NBS, and PPPs + 5% NBS groups (31.18 ± 0.01, 34.31 ± 0.04, and 26.35 ± 0.05 mmol/L, respectively). During culture, glucose concentration decreased only modestly in the 5% NBS group, reaching 24.78 ± 0.05 mmol/L at 144 h, whereas a more pronounced decline was observed in the 10% NBS group, which reached 11.32 ± 0.04 mmol/L. The PPPs + 5% NBS group showed an intermediate and sustained decrease, reaching 18.05 ± 0.03 mmol/L at 144 h.

Lactate concentrations increased in all groups, with the PPPs + 5% NBS group showing the greatest increase. At 144 h, lactate concentrations were 8.04 ± 0.01, 11.33 ± 0.02, and 14.79 ± 0.01 mmol/L in the 5% NBS, 10% NBS, and PPPs + 5% NBS groups, respectively. From 24 h onward, lactate concentrations in the PPPs + 5% NBS group remained higher than those in both control groups. Overall, the three culture conditions exhibited distinct glucose and lactate concentration profiles. However, because the data were not corrected for medium replacement or normalized to cell density, these differences should not be interpreted directly as cumulative substrate consumption, metabolite production, or cell‐specific metabolic rates.

### 3.10 Effect of PPPs on Cell Growth Kinetics

The logistic growth model was fitted to the experimental growth data, and the estimated parameters are presented in Table 5. The R^2^ values were 0.7481, 0.8909, and 0.8878 for the 5% NBS, 10% NBS, and PPPs + 5% NBS groups, respectively. The estimated *μ*_max_ values were 0.0398, 0.0416, and 0.0406 h^−1^, while the corresponding *X*_max_ values were 2.666 × 10^5^, 3.494 × 10^5^, and 3.519 × 10^5^ cells/mL, respectively. The estimated *μ*_max_ and *X*_max_ values of the PPPs + 5% NBS group were closer to those of the 10% NBS control than to those of the 5% NBS control. These results provide a descriptive comparison of the fitted growth patterns among the three culture conditions.

**TABLE 5:** Kinetics model parameter of MDCK cells in different culture conditions.

Fit parameters	Groups		
	5% NBS	10% NBS	1 g/L PPPs + 5% NBS
μ_max_ (h^−1^)	0.0398	0.0416	0.0406
*X*_max_ (10^5^cells/mL)	2.666	3.494	3.519
*R* ^2^	0.7481	0.8909	0.8878

*Note:* *μ*_max_ represents the maximum specific growth rate, *X*_max_ represents the estimated maximum cell density, and *R* ^2^ represents the coefficient of determination describing the goodness of fit of the logistic model.

### 3.11 Effect of PPPs on Short‐Term Cell Cryopreservation and Resuscitation

As illustrated in Figure 7, microscopic observations revealed that when MDCK cells were cultured for 24 h after resuscitation, the cell outlines were clear and distinct, indicating a good growth state of the cells. By the time they were cultured for 48 h, the MDCK cells had essentially grown to cover the surface of the cell flask, forming a tight and dense monolayer, with the viable cell density reaching over 90%. It could be concluded that MDCK cells cultured with a medium containing PPPs maintained their viability and normal proliferation after short‐term cryopreservation and resuscitation.

**FIGURE 7:**
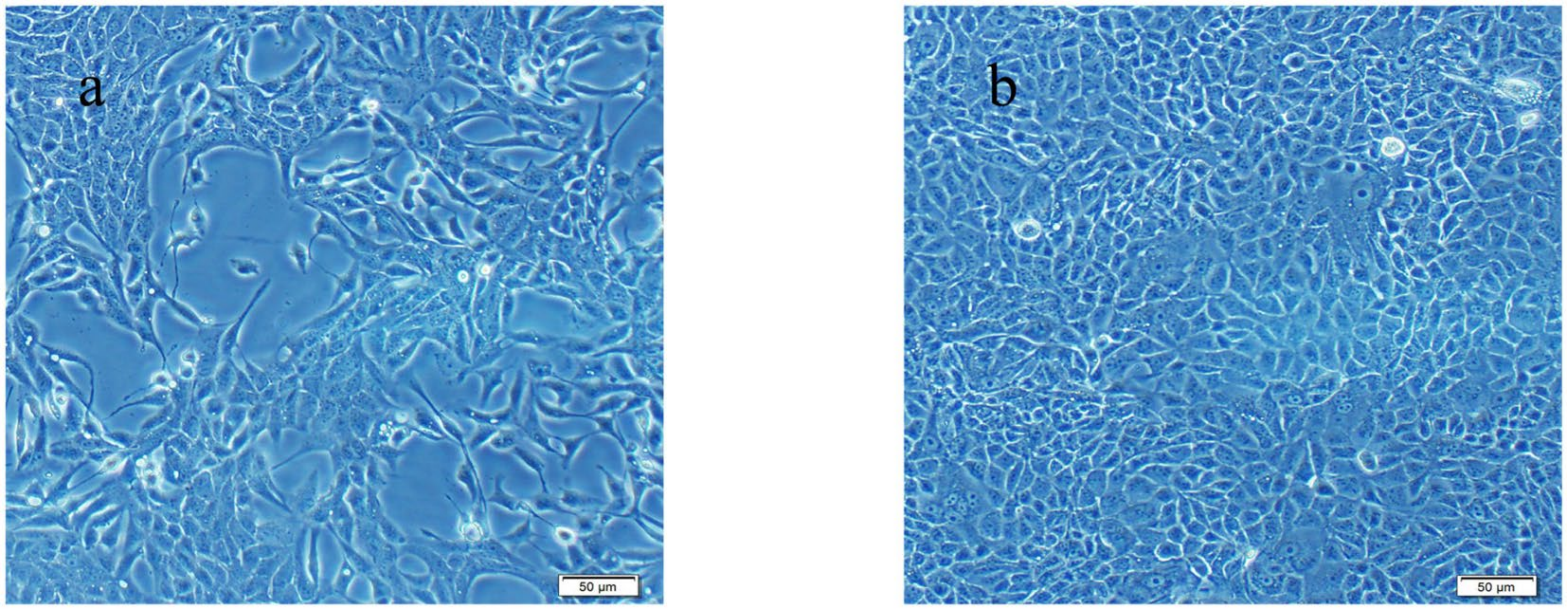
Growth of MDCK adherent cells cultured with PPPs. (a) 24 h; (b) 48 h.

### 3.12 The Efficacy of PPPs in Partially Replacing Serum for Cell Cryopreservation

As shown in Figure 8, the morphology of MDCK cells from the control and experimental groups was examined at 24 h and 48 h post‐thaw. The control group exhibited few dead cells and formed a tightly packed, well‐defined cell monolayer by 48 h. Experimental groups C, D, and E showed comparable cell size and shape post‐thaw. Group E demonstrated strong cell adhesion, reaching approximately 50% cell density at 24 h and developing a dense, compact monolayer by 48 h. Cells in Groups C and D displayed even healthier morphology, with full cell bodies and tight arrangement at 48 h; notably, Group D was morphologically indistinguishable from the control group. In contrast, Group B showed low cell density at 24 h, slow proliferation at 48 h, and scattered, stretched‐out cell morphology. Group A exhibited extremely low cell density at 24 h with severely impaired adhesion; only sparse cells were observed, accompanied by abundant dead cells at 48 h.

**FIGURE 8:**
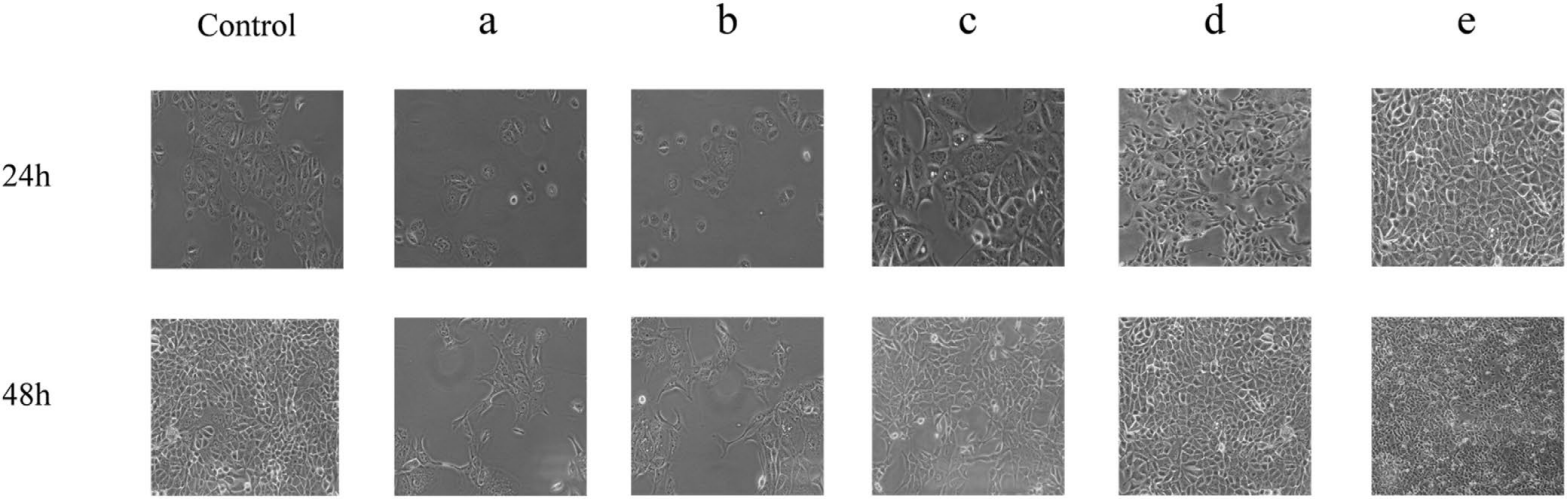
Morphological observation of cells after cryopreservation. (Control): 10% DMSO + 20% NBS + 70% DMEM cryopreservation solution; (a) 10% DMSO + 0% NBS + 90% PPPs medium; (b) 10% DMSO + 5% NBS + 85% PPPs medium; (c) 10% DMSO + 10% NBS + 80% PPPs medium; (d) 10% DMSO + 15% NBS + 75% PPPs medium; (e) 10% DMSO + 20% NBS + 70% PPPs medium.

These observations indicate that PPPs alone cannot fully substitute for serum during short‐term cryopreservation of MDCK cells. Under our experimental conditions, PPPs can partially reduce serum requirements in MDCK cell cryopreservation media; however, a certain amount of serum must be retained in the cryoprotective formulation to achieve satisfactory post‐thaw cell recovery.

## 4 Discussion

The development of serum‐reducing supplements and serum substitutes has become an important research focus in mammalian cell culture, aiming to reduce the costs, batch‐to‐batch variability, and potential biosafety risks associated with animal‐derived serum (van der Valk et al. 2018). Among the alternatives investigated, protein hydrolysates have shown considerable potential because they contain peptides, free amino acids, and other components that may support cell growth. Girón‐Calle et al. (2008) demonstrated the potential of chickpea protein hydrolysates as serum substitutes in THP‐1 cell cultures, while Zhang et al. (2019) reported that silk sericin hydrolysate supported the proliferation of CHO and HeLa cells. Spearman et al. (2014) further demonstrated that the effects of protein hydrolysates on CHO cells varied depending on their composition. In cryopreservation, George et al. (2006) investigated plant‐derived peptones as alternatives to animal proteins in bovine embryo freezing media. Collectively, these studies provide a rationale for exploring plant‐derived protein hydrolysates as serum‐reducing supplements.

Potato protein was selected in the present study because it is an abundant plant‐derived protein resource that can be enzymatically converted into peptide‐rich hydrolysates (Waglay et al. 2014). Chang et al. (2020) demonstrated that enzymatic hydrolysis altered the physicochemical and antioxidant properties of potato protein isolate, supporting further investigation of potato protein hydrolysates as functional biomaterials. In the present study, Flavorzyme hydrolysis yielded PPPs with a degree of hydrolysis of 42.83%. SDS‐PAGE analysis revealed a marked reduction in high‐molecular‐weight protein bands, indicating the degradation of the parent proteins. Furthermore, LC–MS/MS analysis identified 101 peptide sequences, of which 66 (65.35%) had molecular weights between 500 and 1000 Da. Free amino acid analysis also detected 24 amino acid species. Collectively, these findings suggest that the prepared PPPs constitute a heterogeneous hydrolysate containing low‐molecular‐weight peptides and free amino acids. Such compositional characteristics provide a basis for further investigating the potential of PPPs as serum‐reducing supplements for mammalian cell culture.

MDCK cells were selected as the experimental model because of their characteristic epithelial morphology, adherent growth behavior, and established application in influenza vaccine production (Rindler et al. 1979). Therefore, developing culture strategies that maintain MDCK cell growth while reducing serum supplementation is important for improving the consistency of vaccine‐related cell culture processes and reducing their dependence on animal‐derived components. Previous studies have demonstrated the feasibility of culturing MDCK cells under reduced‐serum or serum‐free conditions. Hu et al. (2011) successfully produced inactivated H5N1 influenza vaccines using MDCK cells cultured in serum‐free medium, demonstrating the potential of optimized serum‐free culture systems for vaccine production. Mazurkova et al. (2009) investigated media based on rice and soybean protein hydrolysates and reported only minor changes in the proliferation and morphological characteristics of MDCK cells cultured with 2% FBS. More recently, Cai et al. (2024) established a low‐serum MDCK culture system containing 3% NBS and demonstrated stable cell growth and influenza virus production during serial passaging. These studies indicate that MDCK cells can maintain favorable growth characteristics under reduced‐serum conditions when appropriate culture media are provided. In the present study, characterized PPPs were added as supplements to DMEM containing 5% NBS. By comparing this formulation with 10% NBS and 5% NBS controls, we investigated whether PPPs could partially compensate for reduced serum supplementation while maintaining favorable MDCK cell growth characteristics.

The effectiveness of serum‐reducing supplements depends on their ability to maintain cell growth under different culture conditions and during serial passaging. In the present study, PPPs exhibited concentration‐dependent effects on MDCK cells, with 1 g/L providing the most favorable proliferation and morphological characteristics. Cells supplemented with 0.5–2 g/L PPPs generally maintained compact epithelial morphology, clearly defined cell boundaries, and relatively uniform monolayer formation. In contrast, higher PPPs concentrations (3–5 g/L) were associated with cellular elongation, aggregation, reduced monolayer coverage, and decreased cell density. Previous studies have reported that the biological effects of protein hydrolysates on mammalian cells depend on their composition and concentration. Gupta et al. (2015) demonstrated that the protein and carbohydrate contents of soybean protein hydrolysates influenced animal cell density and IgG production, while Spearman et al. (2014) reported differences in growth‐promoting activity among distinct hydrolysate fractions. These findings suggest that the biological effects of protein hydrolysates are influenced by both their composition and the concentrations of their constituent fractions. Therefore, the reduced cell growth observed at higher PPPs concentrations may reflect the combined effects of different peptide constituents or changes in the physicochemical properties of the culture medium, although these possibilities require further investigation.

To determine whether the growth‐supporting effects of PPPs could be maintained beyond a single culture cycle, MDCK cells were evaluated at passages 3, 5, 7, and 9. At passage 9, the PPPs + 5% NBS group reached a maximum CCK‐8‐derived estimated cell density of 9.11 × 10^4^ cells/mL, with an estimated doubling time of 15.71 h and a specific growth rate of 0.0163 ± 0.0002 h^−1^. The growth characteristics of this group were generally more favorable than those of the 5% NBS control and approached those of the 10% NBS control. These findings suggest that PPPs supplementation helped maintain MDCK cell proliferation during serial passaging under reduced‐serum conditions, consistent with the feasibility of low‐serum MDCK cultivation reported by Cai et al. (2024). The CCK‐8 calibration curve was established under the 10% NBS condition and applied to the other culture conditions. Because CCK‐8 absorbance reflects cellular reducing activity as well as cell number, the derived cell‐density and kinetic parameters should be interpreted as approximate indicators of relative growth rather than precise absolute measurements. Future studies should establish condition‐specific calibration curves or validate these estimates by direct cell counting.

Beyond maintaining proliferation during serial passaging, an effective serum‐reducing supplement should support cell growth under different culture conditions without adversely affecting cellular behavior. Colony‐forming efficiency reflects the ability of individual cells to establish proliferating colonies under low‐density culture conditions (Franken et al. 2006). In the present study, the PPPs + 5% NBS group exhibited a colony‐forming efficiency of 45.1%, which was higher than those of the 10% NBS (43.6%) and 5% NBS (39.8%) controls. This modest increase suggests that PPPs supplementation may support colony formation under reduced‐serum conditions. This observation is broadly consistent with the findings of Xu et al. (2019), who reported that soybean hydrolysate enhanced single‐cell cloning efficiency in CHO‐K1 cultures. Changes in extracellular metabolite concentrations can also provide complementary information on cell culture conditions (Carinhas et al. 2016). Accordingly, glucose and lactate concentrations in the culture medium were monitored in this study. PPPs supplementation was associated with distinct extracellular glucose and lactate concentration profiles. However, because fresh medium was replenished during sampling, these measurements represent time‐point‐specific concentrations and cannot be directly interpreted as cumulative substrate consumption or metabolite production rates. In addition, a logistic growth model was employed to characterize the overall growth patterns under different culture conditions. The fitted growth parameters of the PPPs + 5% NBS group were closer to those of the 10% NBS control than to those of the 5% NBS control. Collectively, these complementary findings suggest that supplementation with an appropriate concentration of PPPs can support favorable MDCK cell growth characteristics under reduced‐serum conditions, although the mechanisms underlying their concentration‐dependent effects remain to be elucidated.

In addition to supporting cell growth during routine cultivation, reducing serum dependence in cryopreservation media is another important objective in the development of animal cell culture systems. Conventional cryopreservation formulations frequently contain serum and DMSO, and alternative biological additives have been investigated to reduce reliance on animal‐derived serum components (Sasaki et al. 2005). George et al. (2006) investigated plant‐derived peptones in animal‐protein‐free freezing media for bovine embryos, demonstrating the feasibility of incorporating plant‐derived components into cryopreservation formulations. Similarly, Varma et al. (2015) evaluated buffalo ocular fluid as a serum replacement in cryopreservation media for different cell types, highlighting the potential of alternative biological materials in cell preservation. In the present study, MDCK cells subjected to PPPs‐associated culture and cryopreservation conditions retained recognizable epithelial morphology and recovered their attachment and proliferation after thawing. Formulations combining PPPs with residual NBS supported more favorable post‐thaw recovery than the serum‐free PPPs formulation, which exhibited poor cell attachment and limited proliferation. These observations suggest that PPPs alone were insufficient to replace serum in the cryopreservation system tested, whereas their combination with residual serum may help maintain post‐thaw cell recovery. Nevertheless, because the present evaluation relied primarily on microscopic observations of cell morphology and attachment, whether PPPs directly mitigate freezing‐induced cellular damage remains unclear.

Overall, this study evaluated the potential of PPPs for reduced‐serum MDCK cell culture through compositional characterization, serial‐passage experiments, colony‐formation assays, extracellular metabolite measurements, growth kinetic modeling, and post‐thaw recovery observations. These complementary assessments provide evidence supporting the potential application of PPPs as serum‐reducing supplements in MDCK cell culture. Nevertheless, further quantitative validation and mechanistic investigations are required. Future studies should focus on identifying the bioactive peptide fractions of PPPs and elucidating the mechanisms underlying their concentration‐dependent effects on MDCK cell growth. Direct cell counting, quantitative metabolic analyses, and comprehensive post‐thaw recovery assessments should also be conducted to further characterize their effects on cell proliferation, metabolism, and cryopreservation performance. In addition, future studies should include appropriate commercial low‐serum or serum‐free media as reference controls to evaluate the practical efficiency and applicability of PPPs‐supplemented media. And the ability of PPPs to support long‐term MDCK cell growth under further reduced‐serum or completely serum‐free conditions should be investigated. Given the importance of MDCK cells in influenza vaccine production, future research should also evaluate the effects of PPPs on viral replication and antigen yield. Furthermore, standardization of PPPs preparation and assessment of batch‐to‐batch consistency will be important for supporting their potential application in low‐serum cell culture and cryopreservation systems.

## 5 Conclusion

This study identified 1 g/L as the optimal PPPs concentration for supporting MDCK cell growth under reduced‐serum conditions. At this concentration, PPPs improved cell proliferation and maintained favorable clonogenic and morphological characteristics. In cryopreservation experiments, PPPs‐containing formulations with reduced serum supported satisfactory post‐thaw recovery, indicating that PPPs may help reduce serum requirements in MDCK cell culture and cryopreservation. Overall, PPPs show potential as serum‐reducing supplements for MDCK cell culture and cryopreservation. Further studies are needed to establish their mechanisms of action, long‐term culture performance, and suitability for vaccine‐related applications.

## Author Contributions

**Dandan Gao:** conceptualization, supervision, writing – review and editing, validation. **Jiwen Wang:** methodology, visualization, writing – original draft, investigation. **Huaxin Li:** validation, investigation, writing – review and editing. **Qianmin Yang:** validation, investigation. **Chenchen Wang:** validation, investigation, formal analysis. **Yujie Cao:** validation. **Junyao Mei:** validation, software. **Xuankang Yang:** validation, software. **Qianwen Bai:** methodology, writing – original draft, data curation. **Gongtao Ding:** project administration, resources, funding acquisition, supervision.

## Funding

This work was supported by Fundamental Research Funds for the Central Universities, 31920250002; Gansu Provincial Department of Science and Technology, 24YFFA026; Gansu Provincial Department of Education, 2026CYZC‐009.

## Ethics Statement

The authors have nothing to report.

## Conflicts of Interest

The authors declare no conflicts of interest.

## Data Availability

The data that support the findings of this study are available from the corresponding author upon reasonable request.
